# Conflicting Views on Chemical Carcinogenesis Arising from the Design and Evaluation of Rodent Carcinogenicity Studies

**DOI:** 10.1289/ehp.9989

**Published:** 2007-11-07

**Authors:** Ronald L. Melnick, Kristina A. Thayer, John R. Bucher

**Affiliations:** National Institute of Environmental Health Sciences, National Institutes of Health, Department of Health and Human Services, Research Triangle Park, North Carolina, USA

**Keywords:** dose selection, maximally tolerated dose, mode of action, rodent cancer bioassay, statistical power, tumor pathology

## Abstract

Conflicting views have been expressed frequently on assessments of human cancer risk of environmental agents based on animal carcinogenicity data; this is primarily because of uncertainties associated with extrapolations of toxicologic findings from studies in experimental animals to human circumstances. Underlying these uncertainties are issues related to how experiments are designed, how rigorously hypotheses are tested, and to what extent assertions extend beyond actual findings. National and international health agencies regard carcinogenicity findings in well-conducted experimental animal studies as evidence of potential carcinogenic risk to humans. Controversies arise when both positive and negative carcinogenicity data exist for a specific agent or when incomplete mechanistic data suggest a possible species difference in response. Issues of experimental design and evaluation that might contribute to disparate results are addressed in this article. To serve as reliable sources of data for the evaluation of the carcinogenic potential of environmental agents, experimental studies must include *a*) animal models that are sensitive to the end points under investigation; *b*) detailed characterization of the agent and the administered doses; *c*) challenging doses and durations of exposure (at least 2 years for rats and mice); *d*) sufficient numbers of animals per dose group to be capable of detecting a true effect; *e*) multiple dose groups to allow characterization of dose–response relationships, *f*) complete and peer-reviewed histopathologic evaluations; and *g*) pairwise comparisons and analyses of trends based on survival-adjusted tumor incidence. Pharmacokinetic models and mechanistic hypotheses may provide insights into the biological behavior of the agent; however, they must be adequately tested before being used to evaluate human cancer risk.

Over the past several years, problems related to conflicts of interest in peer review have received considerable attention in the scientific literature and national press (Harris and Berenson 2005; Steinbrook 2005; Waldman 2005). In response, several scientific journals and international agencies overseeing expert scientific review panels have added stringent rules to reveal real or apparent financial conflicts of interest by individuals or commercial entities (Cogliano et al. 2004). The determination of a real or apparent conflict of interest may result in limitation or disqualification of individuals from participation on expert panels.

In contrast to these circumstances, conflicts of interest are an inherent component of science-based litigation and generally include presentations and interpretations of studies that are fashioned to appear consistent and favorable with the position of the sponsor. This situation puts an enormous burden on judges and juries, forcing them to wade through disguised biases in order to decipher assertions from facts. Because of uncertainties in extrapolations of toxicologic findings from studies in experimental animals to human risk and uncertainties in the costs associated with reduction or elimination of human exposures to those agents, numerous conflicts have arisen and continue to arise over the reliability of identified health effects of specific substances. In one view, precautionary health measures to prevent disease are advocated in spite of uncertainties of the magnitude of potential human risks, whereas the alternative perspective argues that additional costs for exposure reduction are not warranted until adverse health effects are clearly demonstrated in humans. Conflicting views on the relative importance of toxicologic research seem to originate largely from concerns of predictability and impacts on human health risks versus impacts on costs and profits.

Although science seeks to expand our knowledge of facts and truths through the principles of hypothesis generation and hypothesis testing, the way in which our knowledge grows and reflects the truth depends on how questions are framed, how rigorously hypotheses are tested, and to what extent assertions extend beyond actual findings and are portrayed as established facts. Consequently, there are several ways in which conflicting views may arise in health effects research. Failure to adequately test hypotheses or speculations with appropriately challenging experiments does not advance science and, more important, can produce erroneous opinions of potential health effects of particular agents. Reliance on untested hypotheses that are promoted to explain away adverse outcomes may have serious public health consequences if future testing of alleged mechanisms shows them to be incorrect (Tomatis 2002). This article focuses on principles of design and evaluation of animal carcinogenicity studies, their utility for determining disease causality, and potential sources of conflicting views on chemical carcinogenesis.

## Use of Animal Studies for Public Health Decisions

There are several advantages and disadvantages in assessing human cancer risk from animal studies. Animal models are used in preclinical trials of new pharmaceutical agents before testing in humans because of species similarities in the biology of disease processes. The same predictive value of experimental animal studies has been applied to assessments of potential toxic and carcinogenic agents in our environment. A major advantage of animal studies is the elimination of the need to wait for a high incidence of human cancers (which may clinically manifest as much as 30 years from time of first exposure) before implementing public health–protective strategies. Because exposure conditions can be finely controlled in animal studies, they are easier to interpret and assign causality. In contrast, retrospective epidemiology studies typically have limited exposure information, especially at times early in tumor development, and confounding factors are not always known.

Animal carcinogenicity studies can be performed in less time and at lower costs than epidemiology studies. The major disadvantages of animal studies are that they require extrapolations across species and dose. Furthermore, animal studies do not capture the full range of human variability due to differences in genetics, health status, diet, lifestyle, and other exposures.

Because all known human carcinogens that have been studied adequately in experimental animals produced positive carcinogenic results, public health agencies, including the International Agency for Research on Cancer (IARC 2000), the National Toxicology Program (NTP 2004), and the U.S. Environmental Protection Agency (U.S. EPA 2005), have endorsed the perspective that

in the absence of adequate data in humans, it is biologically plausible and prudent to regard agents and mixtures for which there is sufficient evidence of carcinogenicity in experimental animals as if they presented a carcinogenic risk to humans. (IARC 2000)

No alternative experimental approach has been shown to be as reliable for assessing human cancer risk (Tomatis et al. 2001). Hence, even in the absence of adequate human data, public health agencies have classified agents as possibly/probably (IARC 2000), likely (U.S. EPA 2005), or reasonably anticipated (NTP 2004) to be a human carcinogen if there is sufficient evidence in animals. Sufficient evidence includes *a*) an increased incidence of malignant or malignant and benign tumors combined in two or more species or at multiple sites, *b*) an increased incidence in two or more independent studies in one species, or *c*) an increased incidence in a single study in one species if malignant tumors occur to an unusual degree in incidence, site, type, or age of onset. As noted below, mechanistic data on relevant biological activities of the substance can also influence the overall cancer risk classification.

## Experimental Design Issues

The outcome of an animal carcinogenicity study may be affected by several experimental design factors. Differences in experimental design can lead to inconsistent results and conflicting views on the potential health effects of the agent under study. For example, early studies on benzene failed to detect carcinogenic effects in animals, even though epidemiology studies had demonstrated a causal association between benzene exposure and leukemia in humans. Deficiencies in the early animal studies included too few animals, lack of controls, short study duration, and inadequate levels of exposure. Subsequent, better-designed studies by Maltoni et al. (1989) and by Huff et al. (1989) established benzene as a potent, multisite carcinogen in rats and mice. Some experimental design issues that can lead to conflicting results and interpretations on the carcinogenicity activity of a chemical are discussed below.

### Chemical

To best understand the toxicologic properties of a particular agent, the chemical should be tested at high purity. This ensures that the agent under study is responsible for any observed effects and that contaminants are not the cause or modifier of that response. When a potentially active contaminant is present, claims are frequently made that the contaminant and not the principal agent is responsible for any observed carcinogenic response. For example, when the hepatocarcinogenicity of industrial-grade trichloroethylene (TCE) in mice was reported by the National Cancer Institute (1976), Henschler et al. (1977) suggested that the stabilizer epichlorohydrin and not TCE was the causal agent. However, a subsequent study of TCE without epichlorohydrin produced a similar response, confirming the carcinogenicity of TCE (NTP 1990). Resolving all questions about the observed adverse health effects of environmental pollutants such as TCE is time consuming and costly. Health agencies should not allow these types of assertions to cause delays in actions necessary to reduce human exposures.

Before exposing animals to the agent, it is necessary to ascertain the stability and exposure uniformity of the chemical under conditions that simulate the conditions of the study (NTP 2006a). If the agent degrades or evaporates during exposure, the accuracy of the targeted administered dose is compromised and degradation products may contribute to any observed response.

### Animal models

Rats and mice are the two species most typically used in cancer bioassays because they have life spans of about 2.5 years and studies of up to 1,000 animals can be performed in reasonably sized animal rooms. Strains of animals used should be ones that have adequate longevity, genetic stability, and few spontaneous diseases that might shorten their life span, mask any chemical-induced effects, or impair metabolism/elimination of the test agent (Rao et al. 1988). It is difficult to detect responses in organs with very high background tumor rates (e.g., interstitial cell tumors of the testis in F344 rats). Both sexes of two species should be used to identify any sex-specific responses and confirm multiple species effects.

A major shortcoming of the rodent cancer bioassay is its limited statistical power to estimate the true response rate (Haseman 1983). Power is the probability of detecting an effect (rejecting the null hypothesis) when an effect exists; it is influenced by the sample size, the background rate, and the magnitude of the true response (Haseman 1984). The power limitation of the bioassay may lead to conflicting views of study results when nonsignificant elevations in incidence are detected in treatment groups of small size. Small sample size was the basis of conflicting views expressed on the significance of tumors found in long-term toxicity studies of dichlorodiphenyltrichloroethane (DDT) in monkeys (Takayama et al. 1999; Tomatis and Huff 2000).

### Exposure

Because of the limited statistical power of the bioassay when group size is only about 50 animals per sex per dose, high doses are necessary to identify potential carcinogenic hazards, whereas multiple dose groups are used to characterize dose–response relationships. The selection of dose levels is a critical aspect of the experimental design and is a major source of conflicting views in the interpretation of study results. Data from prechronic or subchronic studies (4–13 weeks’ duration) are used to estimate the maximally tolerated dose or the minimally toxic dose (MTD).

**Figure 1:**
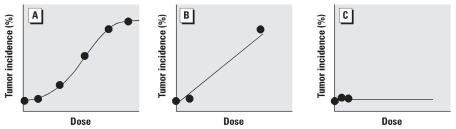
Tumor dose–response curves: (*A*) five dose groups plus control; (*B*) a high-dose group, a much lower dose group, and control; (*C*) two low-dose groups plus control.

Lower doses (1/2 MTD and 1/4 to 1/10 MTD) are used in case the highest dose selected for the chronic study is found to be too high (excessive mortality) and to provide information on dose–response relationships (Bucher et al. 1996). Pharmacokinetic information should also be used to ensure that no more than one of the selected doses is above a level that saturates the processes of absorption, metabolic activation, or detoxification. A cancer bioassay that uses only saturating doses would not be very informative in characterizing dose–response relationships at lower exposures. For example, carcinogenicity studies of 1,3-butadiene in rats were conducted with exposure concentrations of 1,000 and 8,000 ppm (Owen et al. 1987), although metabolism of this gas in rats is linear up to about 1,000 ppm (Bolt et al. 1984). A better characterization of the true dose–response can be achieved with larger numbers of properly spaced dose groups (Figure 1A) (e.g., five-dose study of 1,3-butadiene in mice at 6.25–625 ppm; Melnick et al. 1990) versus only two widely spaced dose groups (Figure 1B). Unless group size is extremely large (i.e., several hundred to thousands of animals per group), the selection of only low doses for the cancer bioassay or short exposure durations can lead to a misinterpretation of the carcinogenic potential of the agent under study and its potential risk at human exposure levels (Figure 1C). For example, chronic studies of *p*-dichlorobenzene failed to detect a carcinogenic effect in rats or mice exposed up to 500 ppm by inhalation for 76 or 57 weeks, respectively (Loeser and Litchfield 1983); in contrast, significant increases in kidney tumors in male rats and liver tumors in male and female mice were observed in 2-year gavage studies of *p*-dichlorobenzene at doses up to 300 or 600 mg/kg, respectively (NTP 1987).

Typical carcinogenicity studies in rats and mice involve exposures beginning at 6 weeks of age and continuing for 2 years; this exposure period corresponds roughly with early adulthood through most of an occupational life span. Earlier periods of exposure should be included if there is reason to believe that susceptibility may be greater during growth and early developmental stages, for example, mutagens (Rice et al. 1989) and endocrine disruptors such as diethylstilbestrol (Herbst et al. 1979). The 2-year duration limit was selected to minimize late-developing background tumor responses in controls and in exposed animals that might preclude the ability to detect significant chemical-induced effects. Exposure durations shorter than 2 years are also problematic because of their reduced sensitivity to detect increases in late-appearing tumors that are related to treatment with the test agent (Haseman et al. 2001).

## Evaluation Issues

The conduct and evaluation of a cancer bioassay require a multidisciplinary effort, including expertise from toxicologists, laboratory animal veterinarians, chemists, histologists, pathologists, cellular/molecular biologists, and statisticians. Topics that affect the interpretation of a properly conducted carcinogenicity study include the thoroughness of the histopathologic evaluations, the statistical analyses, and the appropriate application of mode-of-action hypotheses.

### Histopathology

The detection of nonneoplastic and neoplastic lesions in animals depends on the thoroughness of the necropsies and the microscopic examinations performed. A proper evaluation necessitates that all organs and tissues be examined and that moribund animals be removed from the study and sacrificed immediately to avoid autolytic destruction of cells and tissues (NTP 2006a). Tissue autolysis can interfere with the detection and diagnosis of chemical-induced lesions and, consequently, reduce the power of the study. In some cases, multiple sectioning of an organ in exposed and control groups may be necessary to obtain a more accurate estimate of the incidence of neoplastic lesions, especially for small lesions that may not be detected at necropsy (Eustis et al. 1994). The incidence of renal tubule adenomas was found to be increased significantly in male rats exposed to benzophenone after additional sections of kidneys from control and treated rats were examined microscopically (NTP 2006b). Without the examination of additional sections, this effect might have been missed. Although diagnostic criteria have been established for most observable lesions, it is not unusual for pathologists to disagree in their judgment of lesions, especially those that are part of a continuum of progressive change. Studies that lack independent pathology peer review (Boorman et al. 1985) may yield diagnostic data that would not be generally accepted by rodent pathologists. The use of multiple terms for similar lesions or not properly combining related tumors could lead to underdiagnosis of a chemical-related effect. For example, when the incidences of neuroglial tumors in rats exposed to 1,3-butadiene (Owen et al. 1987) were combined, an additional carcinogenic effect of this chemical became apparent (Melnick and Huff 1992).

In addition to statistical analyses described below, other factors may contribute to the interpretation of tumor data, including *a*) the occurrence of common versus uncommon tumors; *b*) evidence of progression of lesions, such as benign to malignant where it is appropriate to combine (McConnell et al. 1986), or preneoplastic to neoplastic; *c*) tumor occurrence with reduced latency; *d*) multiplicity in a site-specific tumor response; *e*) evidence of metastases; and *f* ) supporting evidence of proliferative preneoplastic lesions at the same site or detection of the same lesion in the other sex or species. Ignoring this type of information could lead to faulty interpretations of chemical-induced carcinogenic effects. For example, a nonstatistically significant increase in urinary bladder tumors in female rats (2 of 49, 4%) is considered to be related to anthraquinone exposure because of the low historical rate of this neoplasm (0.1%) and the marginally increased incidences of hyperplasia of the bladder transitional epithelium (NTP 2005a). Similarly, nonstatistically significant increases in thyroid follicular cell tumors in rats are considered to be related to sodium chlorate exposure because the incidences exceeded historical control rates, and incidences of follicular cell hypertrophy were increased in all exposure groups (NTP 2005b).

### Statistics

If mortality in a dose group is different from that of controls, it is critical that pairwise comparisons and analyses of trends be based on survival-adjusted tumor rates (Haseman 1984). The reason for this adjustment is that if animals died early from causes other than tumors at the organ site of interest, then those animals would not have been on study long enough to provide a full contribution of risk to that study group. Failure to adjust for differences in survival (e.g., Owen et al. 1987) could yield unreliable estimates of cancer risk and possible misinterpretations of a true site-specific effect.

Although comparisons between the concurrent control group and the exposure groups are the most valid for identifying chemical-induced effects, comparisons with historical control data may also be helpful in interpreting treatment-related effects (Haseman et al. 1984). For meaningful comparisons, the conditions of the current study must be similar to those in the historical database and diagnostic criteria must be identical. Thus, comparisons must be specific for the species, sex, and strain of animals and for the route of exposure and the diet. Conflicting interpretations of study findings may arise with improper use of historical databases. For one, the specifications of equivalent study conditions and diagnostic criteria noted above must be followed. For example, although Bird et al. (1997) observed a significant dose-related increase in incidences of testicular tumors in F344 rats exposed to methyl-*t*-butyl ether by inhalation for 2 years (64% controls, 70% at 400 ppm, 82% at 3,000 ppm, and 94% at 8,000 ppm), these findings were dismissed by the authors, as well as by an IARC review panel (IARC 1999a). This decision was based on the perception that the high rate of interstitial cell tumors in untreated male F344 rats makes statistical increases in these rates meaningless, and on claims that the increases in exposed groups were due to a low incidence in the control group compared with historical controls. However, the incidence of testicular tumors in the control group was essentially the same as the historical control rate of testicular tumors in F344 rats in inhalation studies (Nyska et al. 1998). In addition, similar increases in testicular interstitial cell tumors in inhalation studies of ethylbenzene (NTP 1999a) and isoprene (NTP 1999b) in F344 rats were considered related to chemical exposures. In some cases, excessive reliance has been placed on the range of historical response rather than the mean tumor incidence and its measure of variability. This can be problematic if an extreme value in the database contains an outlier value that is not relevant to the current study being evaluated. In such a case, it would be inappropriate to ignore an increased tumor incidence that falls within the historical control range but is much greater (e.g., > 2 SD) than the historical mean control tumor incidence.

### Mechanistic considerations: dosimetry

A critical step in assessing human cancer risk from data obtained in animal studies is to estimate an appropriate human equivalent dose metric. The extrapolation of dose across species is typically performed by scaling in relation to body weight or through the use of physiologically based pharmacokinetic (PBPK) models. PBPK models represent in quantitative terms the complex physiologic and biochemical processes that affect the absorption, distribution, metabolism, and elimination of the agent of concern. These models are developed to describe relationships between exposure to toxic agents, the internalized dose, and the time-dependent tissue concentrations of parent compound and biologically active metabolites. Conflicting views in estimations of human dose may arise because of various assumptions used to determine chronic dose levels by scaling or PBPK modeling. For example, the assumption made in many models that a unique set of metabolic parameters, which in some cases are derived from short exposures of young healthy individuals, can be scaled as a function of body weight to provide precise estimates of the behavior of the agent in genetically diverse populations (e.g., TCE: Clewell et al. 2000; Fisher 2000) is unrealistic.

Additional uncertainties in model parameters arise because multiple isozymes may be involved in the activation or detoxification pathways, and in addition to large differences in enzyme activities due to genetic diversity, individuals may be exposed differently to other agents that can affect the levels of activating or detoxifying enzymes. Simply fitting a model to limited data sets does not provide any assurance that model-based assumptions are reliable. All model assumptions must be made explicit and evaluated for their impact on model predictions. Confirmation of model predictions by subsequent experimentation is essential for hypothesis testing and model validation (Kohn 1995).

### Mechanistic considerations: mode of action

Results from animal or *in vitro* studies that attempt to determine the mode of action for disease induction have been used by public health agencies to upgrade or downgrade cancer risk classifications of agents that have inadequate or limited evidence in humans.

Consider the family of vinyl halides, which includes vinyl chloride (VCl; a known human carcinogen), vinyl bromide (VBr), and vinyl fluoride (VF). These three chemicals are activated by enzymes found in humans (cytochrome P450–dependent monooxygenases, primarily CYP2E1) to epoxides that can react with DNA (Guengerich et al. 1991) to form promutagenic DNA adducts. In animals or *in vitro* systems, the same DNA adducts are formed with VCl, VBr, or VF. Experimental carcinogenicity studies have shown that these three chemicals induce neoplasms of the circulatory system (angiosarcomas), as well as other types of tumors, in rats and mice (Melnick 2002). To consider VBr or VF as anything less than a human carcinogen is unethical. Because VCl is known to cause angiosarcomas in humans, VBr and VF should be classified as human carcinogens without requiring confirming evidence from studies in humans.

Based on mechanistic data, IARC and NTP upgraded ethylene oxide (IARC 1994; NTP 2004) and 2,3,7,8-tetrachlorodibenzo-*p*-dioxin (TCDD) (IARC 1997; NTP 2004) from “probable” or “reasonably anticipated” human carcinogens to “known human carcinogens.” In both cases, the evidence of carcinogenicity was limited in humans and sufficient in experimental animals. The upgrading of ethylene oxide was based largely on results in exposed workers that showed induction of chromosomal aberrations in peripheral lymphocytes, the presence of micronuclei in bone marrow cells, and hemoglobin adducts in blood samples. The upgrading of TCDD was based on data demonstrating that the multi-site carcinogenicity of this chemical in experimental animals was due to a mechanism involving activation of the aryl hydrocarbon receptor and studies showing that this receptor is highly conserved across species and functions the same way in humans and in experimental animals.

On the other hand, IARC downgraded the classification of di(2-ethylhexyl)phthalate (DEHP) from “possibly” to “not classifiable as to its carcinogenicity to humans” (IARC 2000). This was done despite the fact that the evidence of carcinogenicity of DEHP, based on increased incidences of liver tumors in rats and mice, was concluded to be sufficient in animals. Strangely, data on pancreatic tumors induced by DEHP in rats were also available (David et al. 2000) but not reviewed by the IARC working group. The downgrading of the animal cancer evidence was based on the panel’s acceptance of the hypothesis that DEHP induces liver tumors in rats and mice by a non-DNA-reactive mechanism involving peroxisome proliferation. This mechanism was considered not to be relevant to humans because peroxisome proliferation had not been documented either in human hepatocyte cultures exposed to DEHP or in the liver of exposed nonhuman primates. The IARC decision seems unreasonable because peroxisome proliferation alone does not provide a mechanistic explanation for the different carcinogenic potencies of peroxisome proliferators in the rat liver (Marsman et al. 1988).

Peroxisomes are subcellular structures that contain several oxidase enzymes. Agents that cause increases in their numbers are called peroxisome proliferators. Many peroxisome proliferators are rodent carcinogens, and the mode of action proposed for rodent liver tumor induction by peroxisome proliferators involves activation of the peroxisome proliferator–activated receptor (PPARα), which results in altered transcription rates of genes that regulate cell proliferation and apoptosis (programmed cell death) (Klaunig et al. 2003). However, this hypothesis has not been tested with experimental studies demonstrating consistent increases in liver tumor incidence as a direct function of the time-dependent induction of cell proliferation and suppression of apoptosis in rats and mice treated with peroxisome proliferators. An apparent problem with this hypothesis is that increases in cell proliferation are generally only a transient response that returns to control levels within about 2–4 weeks after initiation of continuous exposure, whereas tumor induction requires chronic exposure for most peroxisome proliferators.

Peroxisome proliferation per se does not appear to be a causal event in liver carcinogenesis (Melnick 2001) and therefore may not be a reliable marker for evaluating human cancer risk. Humans express a functionally active PPARα (Sher et al. 1993), and hypolipidemic fibrates modulate lipid homeostasis in humans through activation of this receptor (Schoonjans et al. 1996). Species differences in peroxisome proliferation have been suggested to be due to lower levels of PPARα mRNA in the liver of humans compared with rats and mice (Tugwood et al. 1996). Although stimulation of cell proliferation and suppression of apoptosis have been suggested to favor the proliferation and persistence of DNA-damaged cells that eventually progress to tumors, altered expression of cell growth and apoptosis genes by DEHP has not been demonstrated to be dependent on PPARα activation (Klaunig et al. 2003). In fact, growth factors produced in Kupffer cells by a PPARα-independent pathway are essential for the induced cell proliferation and suppression of apoptosis by peroxisome proliferators (Peters et al. 2000). It is clear that the mechanism of tumor induction by DEHP is not known, and a greater understanding of the interplay between PPARα activation and PPARα-independent Kupffer cell activation is needed. Thus, available mechanistic data do not support IARC’s decision to downgrade DEHP (IARC 2000) based on the hypothesis that liver tumor induction in rats and mice occurs by a mechanism involving peroxisome proliferation that is not relevant to humans. In a recent study, Ito et al. (200) showed that dietary administration of DEHP induces liver tumors in mice lacking a functional PPARα gene. This finding emphasizes the need to test mechanistic hypotheses that, if relied on, might lead to erroneous cancer risk classifications and inadequately protective public health decisions.

## Conclusions

Appropriately conducted animal cancer studies remain the most reliable means of identifying agents that pose a potential human cancer risk. However, as noted above, there are numerous ways in which experimental designs and interpretations can be or have been manipulated or misinterpreted to produce false-negative responses. Studies that use too low doses, too few animals, or too short a duration, as well as evaluations that are based on incomplete necropsy or histopathology, do not combine related tumor effects, fail to adjust for differences in animal survival, or incorrectly use historical control data, would not be expected to produce reliable information on chemical carcinogenesis. It is also important to recognize that rats and mice may be insensitive to certain types of cancers (e.g., prostate) and that timing of exposure may be critical in initiating a carcinogenic response. Thus, the lack of an increased tumor incidence in an adequately conducted 2-year animal study does not necessarily signify that the chemical lacks carcinogenic activity in people.

The observation of consistent dose–response relationships between early cellular and molecular responses and tumor induction in laboratory animals may serve as the basis for developing hypotheses linking the early biological event with the tumor response. However, such hypotheses must be tested rigorously to demonstrate that a causal relationship exists rather than simply a correlation of potentially unlinked events. Similarly, assumptions and predictions of dosimetry models must be validated by experimentation before being used in human risk assessments. Public health decisions that could lead to unrestricted use and exposure to carcinogenic agents should not rely on untested hypotheses (Tomatis 2002).

When high-quality human dose–response data are available, they are used preferentially over animal data to assess human cancer risk (U.S. EPA 2005). However, even when adequate human data are available, animal studies are still valuable in identifying potential data gaps in cancer risks that may not be evident in epidemiologic studies. For example, both acrylamide and 1,3-butadiene induce mammary gland tumors in rodents (Friedman et al. 1995; Melnick and Huff 1992), whereas no association has been reported between occupational exposure to either of these chemicals and breast cancer risk (IARC 1999b; Marsh et al. 1999). Conflicting views on chemical carcinogenesis may arise when animal results differ from human results. In this case the apparent discrepancy may be due simply to the lack of female workers included in the cohort studies of these chemicals. Thus, animal studies can reveal inadequacies in conclusions about potential cancer risks in the general population that are based on occupational cohort mortality studies of healthy male workers.

Similar to animal studies, epidemiologic studies may not detect a significant cancer response if group size is too small or if exposures are of insufficient magnitude or duration. On the basis of cancer risk estimates from animal studies on acrylamide, Dybing and Sanner (2003) concluded that epidemiologic studies on this chemical were not able to detect significant increases in cancer risk because of too few participants in a dietary study (Mucci et al. 2003) and too low cumulative exposures in an occupational cohort mortality study (Marsh et al. 1999).

Differences in exposure scenarios between animals and humans might affect the ability of epidemiologic studies to detect a tumor response identified in animal studies. For example, if gestational or early childhood exposure is important for tumor induction, then studies of male workers would not be expected to produce the same response as animal studies that include exposures during these stages of development. Other reasons why an epidemiologic study may not detect a true increase in risk of certain cancers in exposed human populations include inadequate exposure information, mis-classifications, insufficient follow-up, and/or inadequate study power. Because of the much greater genetic diversity in humans compared with specific strains of laboratory animals, the range of expected human response may include subgroups that are less, equally, or more sensitive than the animals used in the experimental studies. More research is needed to understand and adequately account for factors that might contribute to variability in human susceptibility to chemical carcinogenesis. In the meantime, it is prudent to assume that an induced carcinogenic response in animals is a reliable indicator of potential cancer risk in humans (IARC 2000; NTP 2004; U.S. EPA 2005).
